# Growth Kinetics of Bacterial Pili from Pairwise Pilin Association Rates

**DOI:** 10.1371/journal.pone.0063065

**Published:** 2013-05-07

**Authors:** Diana C. F. Monteiro, Wilfride V. Petnga Kamdoum, Emanuele Paci

**Affiliations:** Astbury Centre for Structural Molecular Biology, University of Leeds, Leeds, United Kingdom; Griffith University, Australia

## Abstract

Bacterial pilogenesis is a remarkable example of biological non-templated self-assembly where a small number of different building blocks are arranged in a specific order resulting in a macroscopic hair-like fiber containing up to thousands copies of protein subunits. A number of advanced experimental techniques have been used to understand pilus growth. While details such as the conformation of the protein building blocks before and after the elementary polymerization step have enhanced our understanding of this mechanism, such information does not explain the high efficiency of this growth process. In this study, we focused on the growth of the *Escherichia coli* P-pilus, which is formed by the assembly of six subunits, structurally similar incomplete Ig-like domains. These subunits undergo polymerization through fold complementation by the donation of a β-sheet strand in a specific conserved order. All pairwise rates of association of the individual subunits with the corresponding β-sheet donor strand peptides have been previously determined through non-covalent mass-spectrometry. Here we use computational simulations to determine donor-strand exchange rates and subunit concentrations necessary to warrant the growth of pili showing similar lengths and subunit orders to those observed *in vivo*. Our findings confirm that additional factors must be involved in the modulation of the donor-strand exchange rate and/or pilin subunit concentration at the usher must be important for the precise ordering and rapid polymerization rates observed *in vivo*.

## Introduction

Gram-negative bacteria rely on hair-like fibers, known as pili, on their surface for attachment to host cells prior to infection. This study focused on the P-pili found in the uropathogenic *Escherichia coli*, which are essential for the attachment of the bacteria to kidney cells. Pili are formed by the non-covalent polymerization of different pilins: small proteins with incomplete Ig-like folds lacking the seven strand (stand G) through C-terminal truncation. As a result, pilins exhibit a hydrophobic groove characterized by five binding pockets, P1 to P5, for the hydrophobic side-chains of the missing strand. After being transported into the periplasm via the SecYEG translocon, subunit folding requires a periplasmic chaperone, which donates, *in trans,* one β-strand, strand G1, occupying pockets P1–P4 and thereby complementing the pilin Ig-like fold. This mode of chaperone function is often referred to as “donor-strand complementation” (DSC) [1], [2]. The chaperone prevents premature subunit polymerization in the periplasm, and delivers the pilin subunits in a polymerization-competent state for future assembly at the outer membrane (reviewed in [3]).

Subunit polymerization into a pilus occurs at a specialized transmembrane assembly platform termed “the usher”. Chaperone:subunit complexes are recruited at the N-terminal domain of the usher. Each subunit contains an N-terminal extension (Nte) of 11–17 amino acids, which, during polymerization, replaces the chaperone donor-strand complementing the Ig-like fold in a mechanism referred to as “donor-strand exchange” (DSE). Experimental evidence has demonstrated that this exchange of donor strands from the chaperone to that of a cognate subunit (one that is adjacent in the natural order of pilus assembly) is a concerted process [4]–[7]. A key factor promoting DSE initiation is P5 pocket availability. It was shown through simulation on the PapF subunit of P pili and on the Saf pilus (an analogous system) that unless the P5 binding pocket is accessible, permanently or by conformational change, no subunit:subunit complex formation can occur.

The six different pilins that polymerize to form a P pili are named Pap*X*, with *X* equal to *G*, *F*, *E*, *K*, *A* and *H*. In the periplasm each pilin is bound to the chaperone (Pap*D*), which prevents their aggregation. The first subunit to be assembled (Pap*G*) has an additional domain, the lectin domain, responsible for the adhesion to glycolipid globoside receptors on kidney cells [8]. The tip of the pilus is composed by one Pap*G* pilin followed by one Pap*F,* five to ten Pap*E* – which provide flexibility – and one Pap*K*. The tip is followed by hundreds to thousands of Pap*A* subunits, which form a rigid superhelical quaternary structure. The growth is halted by the inclusion of Pap*H*, which anchors the pilus and terminates assembly. Pap gene knockout studies have provided insight in to the possible roles of the different subunits within the pilus. It was found that the pilus could still function in the absence of Pap*E* or Pap*K* [9], but not without Pap*F* [10].

The formation of pili has been studied using a broad spectrum of experimental techniques, but many questions still remain unanswered. Here we focus on the more general question of how nature has harnessed a series of simple reactions to assemble large macroscopic structures involving thousands of individual proteins in a specific order. The question is not trivial: if reactions between units were exclusive, then a thousand different immunoglobulin domains, each able to accept only the Nte of another, would be needed for pilus formation. Instead, in the specific case of the P pilus only six are used. If all the DSE reactions were equally probable, the probability of a correctly assembled, sufficiently long pilus would be negligible.

Electrospray ionization mass spectrometry (ESI-MS) has been used to measure the (pseudo first-order) rate of association between the six chaperone-pilin complexes and the five Nte peptides *in vitro* [11]. The results revealed that reactions between “cognate” pairs proceed faster than those between non-cognate pairs, suggesting that differences in the rates of DSE between different subunit types may play a role in helping to determine subunit ordering *in vivo*.

Below we explore, by using a simple mathematical model, a number of scenarios that may be relevant *in vivo*. We propose a set of conditions regarding either the pairwise rates of association or the concentrations of the various subunits at the usher that warrant the growth of functional and sufficiently long pilus, as observed *in vivo*. Such conditions support either a key role of the usher in selectively catalyzing specific DSE reactions or a very inhomogeneous concentration of the different pilins in the periplasm, or at least at the usher-membrane interface, in order to result in an efficient production of hundreds to thousands pili *in vivo*.

## Methods

**Figure 1:**
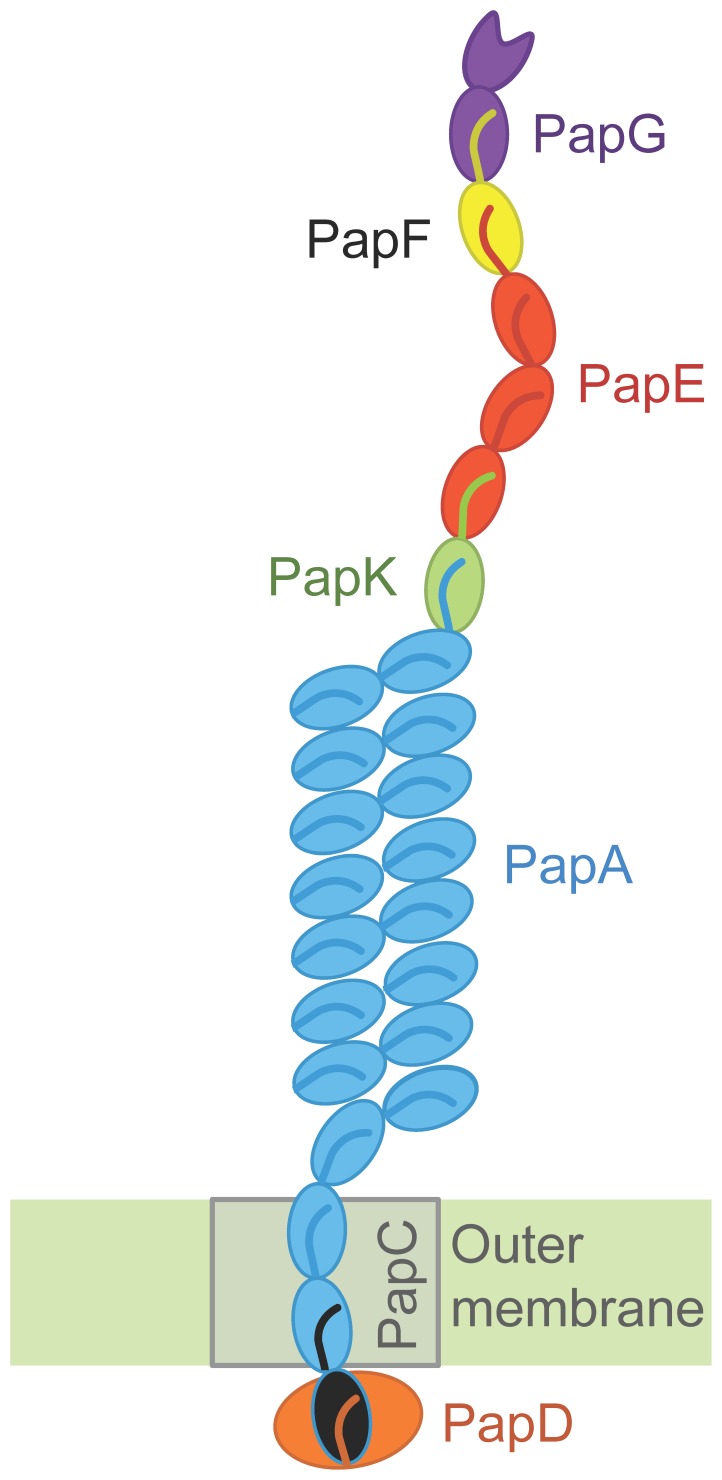
Schematic representation of the P pilus of *E. Coli*. Pap*G* includes an adhesin domain, while the other pilin subunits Pap*F*, Pap*E*, Pap*K*, Pap*A* and Pap*H* have a N-terminal extension the complements the incomplete fold of each subunit. Pap*E* occurs 5–10 times and Pap*A* about 1000 times in natural mature pili. Pap*H* caps the pilus and stops its growth. Pap*C* is the outer membrane usher where assembly occurs *in vivo*, and Pap*D* is the chaperone bound to each pilin subunit in the periplasm.

We define here the quantity:
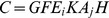
(1)where *i*,*j*≥1, is the “correct” or “naturally occurring” sequence of pilins in a functional pilus (see Figure 1). *P*(*n*) is the probability that a pilus is *n* units long, which is also equal to the probability of finding Pap*H* in position *n*. *P*(*C*,*n*) = *P*(*C*|*n*)*P*(*n*) is the probability that a pilus is “correct” i.e., that its sequence is as Eq. 1, and *n* units long. Another relevant quantity is the probability P* that a pilus has a correct sequence and is longer than n_min_ units:
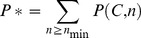
(2)


The growth is simulated by assuming that the basic reaction is [A]+[D]↔[AD] with on-rate 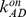 and 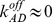; the number and nature of pilins already assembled does not affect the rates above; the concentrations of the individual pilins are not affected by the pilus’ growth (i.e., there is an unlimited amount of each subunit).

With these assumptions, the probability in a short time interval *dt* that a unit *D* will donate its Nte to the end of the growing pilus ending with subunit *A* attached to its chaperone is simply equal to *k_AD_ dt*. We use *k_AD_* rates to generate pili by randomly extracting subunits with a probability proportional to their concentration. With excess concentration of Nte peptides [11], the rate *k_AD_* becomes independent of the concentration of the donor and first order kinetics are observed experimentally. This model could be adapted to fit the more complex second order kinetics observed [12] at lower donor concentrations.

## Results

A considerable number of different experiments have provided valuable insight into the growth of bacterial pili. However, much remains unresolved about how the ordering of subunits and sufficient rate enhancement is achieved by the transmembrane usher in order to describe the specificity and overall rate of pilus biogenesis *in vivo*. We make here a broad number of hypotheses, starting from known uncatalyzed rates of DSE using peptide mimetics of the Nte that we then alternatively release to probe the importance of different factors.

### Varying Pairwise Association Rates

**Figure 2:**
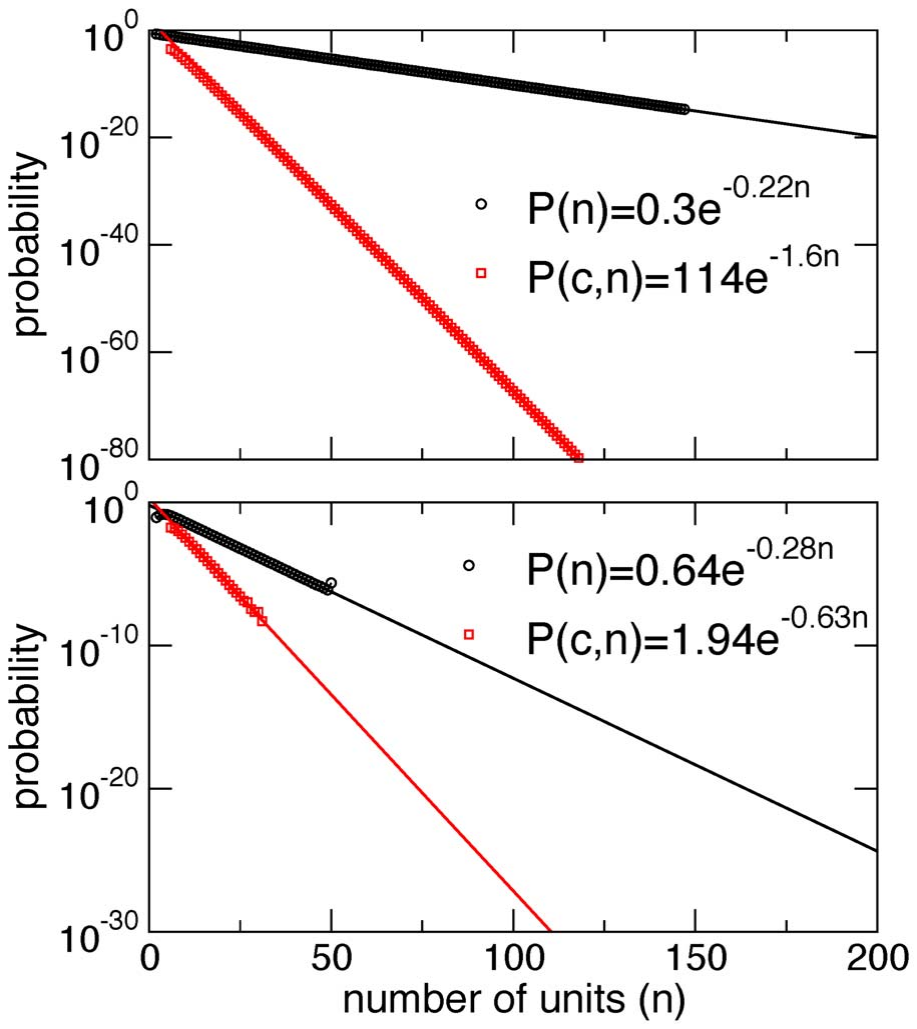
Probability of a pilus *n* units long and probability of a pilus with domains in a correct order and *n* units long. Probabilities decay exponentially and the latter faster than the former. a) Assuming equal concentration and equal rates. b) Assuming equal concentrations and using the experimental rates in Table 2.

We start by assuming that the relative concentration of all subunits is identical in the periplasm, and, more specifically, at the usher: [Pap*G*] = [Pap*F*] = [Pap*E*] = [Pap*K*] = [Pap*A*] = [Pap*H*]. One other assumption, which is certainly incorrect but yet instructive to consider, is that the rate of association between the donor and acceptor is the same for each donor-acceptor pair, except those involving Pap*H* as acceptor and Pap*G* as donor which are taken to be zero (translating for the fact that Pap*H* cannot undergo DSE [5] and that the Nte of Pap*G* is part of the lectin binding domain and not available for DSE). The two conditions above correspond to completely random growth. In such case, both *P*(*n*) and *P*(*C*,*n*) can be computed analytically; all such probabilities decay exponentially for large *n* and are shown in Figure 2a. The fast decay with *n* is not unexpected, and makes it clear why P* is negligible and independent from n_min_ (see Table 1).

**Table 1:** Probabilities P* of a pilus longer than n_min_ and with the correct ordering.

conditions	n_min_ = 100	n_min_ = 1000
equal rates/equal concentrations	3.5 10^−68^	5.8 10^−692^
*in vitro* rates/equal concentrations	1.1 10^−27^	1.2 10^−274^
**R**	1.7 10^−1^	4.8 10^−3^
**C**	4.9 10^−5^	2.6 10^−7^

R refers to rates in Table 3; C refers to the case where the concentration of [A] is 200 times larger than that of any other subunit.

One rapid estimation of a reasonable value of P* can be obtained by considering that each *E. coli* bacterium has of the order of 10^3^ pili and assuming that at least ∼10 pili are necessary for bacterial adhesion [13]: the lowest acceptable probability P* should be ∼10^−2^ = 1%.

Recent measurements of the apparent rates of DSE for each subunit/Nte pair using Nte peptide mimetics and ESI MS [11] have demonstrated that chaperone-subunit complexes react with different Nte peptides with remarkably different rates. Such rates are shown in Table 2. However, despite the apparent discrimination of these rates, with cognate reactions occurring more rapidly than their non-cognate counterparts, the rate differences do not explain the specificity observed *in vivo*. Rates *in vivo* must differ substantially from these calculated *in vitro* [14]–[16]. Other factors, such as the proximity and/or alignment of subunits and at the usher or the presence of the preceding pilin protein as the Nte donor, ought to be involved in pilin polymerization.

**Table 2:** Apparent pseudo first-order rate constants for DSE involving each chaperone/subunit–Nte pair 1000×h^−1^ experimentally measured [11].

	G_Nte_	F_Nte_	E_Nte_	K_Nte_	A_Nte_	H_Nte_
PapD-PapG	0	5.5	∼0.7	∼0.7	∼0.7	∼0.7
PapD-PapF	0	2.7	9.4	4.7	2.8	2.5
PapD-PapE	0	65.7	190.2	210.8	39.9	48.6
PapD-PapK	0	∼1	∼1	1.6	53.6	26.6
PapD-PapA	0	2.9	3.3	9.0	45.8	22.9
PapD-PapH	0	0	0	0	0	0

Simulation of pilus growth using the experimentally determined pseudo first order rates [11] shows a shift toward a much higher probability of long and correct pili; the results are shown in Figure 2b. However, the resulting probabilities P*, based on the exponential fit of the simulation results (Table 1) show that, while such rates indeed amplify significantly the probability P* obtained from random growth, its absolute value is still negligible. In other words, using the relative rate of DSE obtained using peptide mimetics of the Nte *in vitro* cannot recapitulate the probability of successful assembly *in vivo*.

We observe here that from the rates of DSE obtained *in vitro* (in the presence of excess peptide Nte) assembly of a pilus with 100 subdomains takes an average time of ∼2000 hours while pili are observed to grow within minutes *in vivo* [17].

A question naturally arises: is there a choice of rates able to warrant the growth of a correct pilus even retaining the hypothesis that local concentrations of chaperone-bound subunits in proximity of the usher are all equal? To answer this question, we performed simulations using an array of different association rates. An interesting result was obtained using the rates shown in Table 3. In this model, all reactions (except those which involve Pap*G* as donor and Pap*H* as acceptor) occur with finite rate, and those between pairs that are non-cognate in naturally occurring pili occur at a rate 1000 times slower (k_slow_ = k_fast_/1000). In such a case, the probability P* of a long correct pilus is large enough (17% if the minimal length is 100 units and 0.5% if it is 1000 units) to be biologically relevant (see case **R** in Table 1). The factor 1000 between fast and slow rates was chosen by trial and error; if a factor of only 100 is assumed then P* ∼10^−18^ is obtained instead. Another key factor is that the rate of self-polymerization of Pap*A* must be large (k_AA_>1000k_slow_) to allow for the formation of the large helical central stretch of the pilus; results similar to those above are obtained if the DSE rate between Pap*E* and Pap*E* is assumed to be slow (k_EE_ = k_slow_).

**Table 3:** Hypothetical rates which warrant growth of pili where the domains are correctly ordered even if concentrations are all equal.

	G_Nte_	F_Nte_	E_Nte_	K_Nte_	A_Nte_	H_Nte_
PapD-PapG	0	1000	1	1	1	1
PapD-PapF	0	1	1000	1	1	1
PapD-PapE	0	1	1000	1000	1	1
PapD-PapK	0	1	1	1	1000	1
PapD-PapA	0	1	1	1	1000	1
PapD-PapH	0	0	0	0	0	0

### Varying Relative Concentrations

An important assumption in all the results described so far is that the relative concentration of all the chaperone:subunit complexes is equal in the periplasm. Since, as we noted above, naturally occurring pili are mainly composed of Pap*A*, we altered the relative concentration of Pap*A* (i.e., the probability that at each Monte Carlo cycle Pap*A* is randomly picked) by a factor ranging from 2 to 200. If the concentration of Pap*A* is 200 times larger than each of the other subunits the probability P* becomes a reasonable number (1.4% if the minimal length is 100 units and 0.22% if it is 1000 units; see case **C** in Table 1). Decreasing the concentration of Pap*H* relative to the other subunits is also effective in improving the growth of long and correct pili. Indeed, if one assumes that [Pap*A*] is at least 6 times larger than [Pap*E*], [Pap*F*] and [Pap*K*] and [Pap*H*] 10 times smaller than then the growth of a few functionally long pili becomes likely. We stress that while a high [Pap*A*] together with the experimental rates previously determined experimentally [11] may warrant growth of functional pili, the rates obtained are still anomalously slow. This is fully in accord with the previously illustrated catalytic effect of the usher [15]. In a case of very non-uniform concentrations, the usher may simply amplify all the pairwise rates by the same factor (about 10^5^ for growth within minutes). An alternative solution would be that DSE with the Nte as the N-terminus of the pilin subunit occurs with rates significantly faster than the ones observed in vitro with Nte peptide mimetics.

## Discussion

Pilus growth is a fascinating example of biological self-organization where a small number of protein subunits polymerize to form macroscopic structures with high fidelity. For pili, the order of the subunits determines both the biological function of the pilus and its physical properties [18]–[20]. *In vitro* pairwise association pseudo first order rates between all possible subunits and Nte peptides reveal a remarkable preference for “cognate” association [11]. We have shown here that *in vitro* rates alone cannot explain the growth rate or the specificity of subunits’ sequence in the pili assembled *in vivo* at the usher platform.

The observation that pilus formation occurs more rapidly *in vivo* (minute timescale) [17], compared with the much slower timescale of DSE observed *in vitro*, is consistent with the known active role of the usher in subunit polymerization. Indeed, recent experiments have shown that the Fim*D* usher of Type I pili (structurally similar to P pili) acts as a catalyst for DSE [15]. The usher is thought to facilitate assembly of the pilins into pili fibrils in two ways. First, by promoting the dissociation of the chaperone:pilin complex during DSE. Second, by using a hand-over mechanism to translocate newly recruited chaperone-subunit complex bound at the N-terminal domain (NTD) to either part of its C-terminal domain (CTD1 or CTD2) by the intermediary of the usher-plug while a new incoming cognate chaperone:subunit complex binds to the NTD. This new arrangement places the N-terminal extension of the subunit at the NTD directly above the P5 binding pocket of the subunit bound at the CTD, encouraging DSE [21]–[24]. Our results also show that the usher recognizes cognate subunit:Nte pairs over their non-cognate counterparts and boosts their association rate by a factor of 100 or more. This discriminative role played by the usher was suggested by Morrissey *et al.* [22] after evidence showed that the usher’s affinity for chaperone:subunit complexes (Pap*D*-Pap*X*) decreases in accordance with the subunit’s position within the pilus.

The relative periplasmic concentrations of different subunits and the complementarity of the interaction between different Ntes and the acceptor grooves has also been suggested as other possible important factors for DSE [25]. Here we have not considered the possibility that association between particular pairs is reversible because recent evidence strongly suggests that subunit-subunit complexes are kinetically stable against dissociation. The present results show that the occurrence of different subunits with substantial different frequency could warrant the correct growth of long pili, assuming that the *in vitro* rates emulate those *in vivo* without any effect of the usher and also that the concentration of *A* is at least 200 times larger than that of the other pilins.

Using a simple numerical model and some *in vitro* pairwise DSE rates we were able to identify the factors needed to kinetically control correct pilogenesis. We found that with the determined association rates between pilins *in vitro* for subunit:Nte pairs, the relative concentration of pilins in the periplasm would have to be highly conditioned to allow for the growth of functional pili, which would, however, occur at much slower rates than those observed *in vivo.* If instead pilin concentrations in the periplasm were similar for all subunits, then the rates for some DSE reactions would have to differ by up to 1000 fold. Indeed, an intermediate situation is also possible, where both the DSE rates are affected by the usher and the relative subunit concentrations are modulated in the periplasm. The present study shows exactly how much the combined effect should be, thus facilitating the discovery of the factors involved in promoting these effects. Such factors may lie in the SecYEG secretory pathway used to translocate individual pilins across the inner membrane, its affinity for each pilin or the stability of individual pilins after expression and their intrinsic tendency to aggregate.

At lower donor concentrations, more complex second order DSE kinetics has been observed for a few subunits of the analogous Pap pilus [12]. The approach presented here could be easily adapted to second order kinetics; however, no complete set of pairwise second order rates have been determined so far either for the Fim pilus or analogous systems, nor the initial concentrations of pilins in the periplasm determined.

An interesting consequence of a growth model based on effective rates of subunit binding and relative pilins concentrations, is that the length distribution of mature pili is exponentially distributed; thus pilus assembly appears highly inefficient since pili too short to be functional would be the most common outcome of the assembly process. One hypothesis is that very few functional pili are needed; another is that an additional mechanism may be in place which controls the length distribution of pili, for example by modulating the catalytic properties of the usher depending on the size of the growing pilus. To the best of our knowledge the length distribution of pili on uropathogenic *E.coli* is not known and it is thus not useful to speculate further of what the origin of specific distributions may be.

Another observation concerns the pseudo first-order rate constants for DSE in Table 2. They show, as previously described [11], that cognate pairs of subunits bind faster than non cognate pairs. But more interestingly, it can be observed that, as a general trend, DSE rates depend on the specific subunit bound to the chaperon more than it depends on the Nte peptide. For example, the Pap*D*-Pap*E* complex has large DSE rates with all Nte peptides, not just with its cognates Pap*E* and Pap*K* Nte peptides, and all binding rates are at least 10 times faster than those observed for the Pap*D*-Pap*F* complex. Analogously, the Pap*D*-Pap*G* complex has a low DSE rate with all Nte peptides.

The fact that DSE rate depends mostly on the chaperon-subunit complexes is in line with previous observations on the importance of the subunit’s P5 pocket in determining the DSE rate. Atomistic simulation showed that Pap*F* binds slowly with any Nte peptide (and only marginally faster with it cognate Nte) because its P5 pocket, obstructed in the crystal structure, is only intermittently available for binding [5]. Pap*H*, on the other hand, has the P5 pocket always obstructed, which explains why it does not undergo DSE and stops the growth.

Thus, there is an additional interesting hypothesis that the present simultaneous analysis of pairwise association rates between the building blocks of *E.coli* P-pili brings about. Pili are made of structurally very similar subunits, all incomplete Ig domains that bind the same chaperon in the periplasm. The chaperon, itself a two-Ig-domain protein, binds at the same hydrophobic pockets where the N-terminal extension of each subunit can bind. This efficient, fail-proof mechanism behind the assembly of pili where thousands of subunits occur in a specific number and order is also parsimonious: only a handful of subunits is needed, all with almost identical structure, that mutually bind through the same mechanism. Here we have shown that this parsimonious strategy has an undesirable consequence: subunits that should not be adjacent in a functional pilus, although mutually bind with a much lower rate than cognate ones, can still occur next to each other with a very large probability during the growth of a pilus (and very likely cause a premature termination of the assembly). A still unknown mechanism, that involves specific, and possibly history-dependent, modulations of the pairwise DSE rates at the usher, or a modulation of the instantaneous relative concentration of subunits at the usher, must be in place to insure the growth of functional pili on the bacterial surface. Understanding of such a mechanism will have important consequences on our ability to impair the bacterial adhesion, and to exploit such an efficient strategy in synthetic biology applications.

In conclusion, we have shown that measurement and systematic analysis of the binding rates between all the different subunits making up a complex structure like a pilus, even in conditions quite different from those *in vivo*, provide an unexpectedly broad and insightful picture that relates the emergence of large assemblies on bacterial surfaces.
